# Andragogy in Practice: Applying a Theoretical Framework to Team Science Training in Biomedical Research

**DOI:** 10.3389/bjbs.2024.12651

**Published:** 2024-03-28

**Authors:** Jacqueline M. Knapke, Laura Hildreth, Jennifer R. Molano, Stephanie M. Schuckman, Jason T. Blackard, Megan Johnstone, Elizabeth J. Kopras, M. K. Lamkin, Rebecca C. Lee, John R. Kues, Angela Mendell

**Affiliations:** ^1^ College of Medicine, University of Cincinnati, Cincinnati, OH, United States; ^2^ College of Nursing, University of Cincinnati, Cincinnati, OH, United States; ^3^ College of Cooperative Education and Professional Studies, University of Cincinnati, Cincinnati, OH, United States

**Keywords:** team science training, biomedical research education, educational theory, andragogy, adult learning

## Abstract

This study is the first to apply the theoretical principles of Malcolm Knowles’ theory of andragogy to evaluate data collected from learners who participated in team science training workshops in a biomedical research setting. Briefly, andragogy includes six principles: the learner’s self-concept, the role of experience, readiness to learn, orientation to learning, the learner’s need to know, and intrinsic motivation. Using an embedded study design, the primary focus was on qualitative data, with quantitative data complementing the qualitative findings. The deductive analysis demonstrated that approximately 85% of the qualitative data could be connected to at least one andragogical principle. Participant responses to positive evaluation questions were largely related to two principles: readiness to learn and problem-based learning orientation. Participant responses to negative questions were largely connected to two different principles: the role of experience and self-direction. Inductive analysis found an additional theme: meeting biological needs. Quantitative survey results supported the qualitative findings. The study findings demonstrate that andragogy can serve as a valuable construct to integrate into the development of effective team science training for biomedical researchers.

## Introduction

Malcolm Knowles developed the theory of andragogy to provide a framework for understanding the distinct learning patterns of adult learners [1, 2]. Knowles’ theory suggests that instructors should understand and attend to the unique aspects of adult learning motivation. Adult learners often balance numerous commitments, and their educational goals are based on well-defined needs [3, 4]. Compared to their younger counterparts, adult learners are frequently more motivated to perform well in their studies and are more oriented towards task completion [5]. In many cases, adult learners choose to advance their education to retain a competitive edge in the workplace, especially in times of economic recession [3]. Table 1 provides a brief summary of each of the six principles of Knowles’ Theory of Andragogy.

**TABLE 1:** The six principles of Knowles’ andragogy theory.

Principle	Description
Self-Concept	As a person matures, their self-concept moves from that of a dependent personality to that of a self-directed human being. Children maintain a self-concept of total dependency, but adulthood is characterised by a self-concept of self-direction. Once this psychological maturation occurs, the adult naturally feels most comfortable in situations that allow him or her to self-direct, in an independent way.
Role of Experience	As people mature, they accumulate a growing reservoir of experience that becomes an increasing resource for learning. Unlike children, who define themselves in terms of other people (teachers, parents, siblings, etc.), adults define themselves by their experiences. Experience, in itself, can be a form of expertise that teachers should draw upon and use as a resource for learning.
Readiness to Learn	As a person matures, their readiness to learn becomes increasingly oriented towards the developmental tasks of their social roles and life situations. Adult learners want to learn because of the roles they play in their current stage of life, whether at work or home as parents or spouses.
Orientation to Learning	Traditional pedagogy assumes that young students have a subject-based approach to learning, partly because they do not have much life experience. Andragogy assumes that adults approach learning with a problem-based approach.
Need to Know/Why	Adults often pursue education because they need to know something. Adult learners carefully consider *why* they are learning something. In pedagogy, it is assumed that students will simply learn what they are told to learn. Adults want to understand what they will do with the information in life and how it will benefit them or be of consequence to them if they do not learn it.
Intrinsic Motivation	Adult learners are responsive to some external motivators (better jobs, promotions, higher salaries), but the most potent motivators are internal pressures (the desire for increased job satisfaction, self-esteem, or quality of life).

Andragogy has been applied to several fields ranging from chemistry [6] to the coaching of “master athlete” swimmers [7]. Moreover, it is useful in several distinct educational fields including physical education [8], early childhood education teacher training [9], outdoor science education [10], police training [11], military education [12], and social work [13]. One study explored the value of an andragogical framework in a study of blended learning among part-time adult learners pursuing vocational degrees through distance learning [14]. Another study performed a randomised controlled trial to compare the effectiveness of the experiential learning principle of andragogy in teaching nutrition concepts to culinary arts students and found that experiential learning was more effective [15].

Although andragogy is a useful theoretical lens in a wide variety of fields, it has rarely been applied to medical and health sciences disciplines. One study incorporated andragogical principles through the use of podcasts in undergraduate kinesiology courses [16]. Another promoted the use of andragogy in online nursing education [17]. A 2012 study suggested incorporating Knowles’ principles of andragogy into the teaching of medical residents [18]. Another study showed that incorporating learner self-direction in the form of a flipped classroom model yielded higher test scores for Emergency Medicine residents over time compared to traditional teaching methods [19]. Knowles promoted the importance of andragogy in the continuing education of health professionals, given the rapid changes in the field and the mandatory nature of professional education [20]. Another paper, however, argues that the andragogical principle of intrinsic motivation is “simplistic, misleading, and counterproductive” when applied to medical students [21]. We found no existing literature applying andragogy in a biomedical research setting. Furthermore, few studies have explored the utility of andragogy from the perspective of the learners themselves.

Since 2016, the Center for Improvement Science (CIS) at the University of Cincinnati (UC) has offered more than 50 presentations and workshops aimed at teaching biomedical research professionals how to better collaborate on research teams. The CIS operates within a “Science of Team Science” environment that is largely driven by our local National Institutes of Health (NIH)/National Center for Advancing Translational Science (NCATS)-funded Clinical & Translational Science Award (CTSA). The Science of Team Science has been defined as “. . . a new interdisciplinary field … which aims to better understand the circumstances that facilitate or hinder effective team-based research and practice to identify the unique outcomes of these approaches in the areas of productivity, innovation, and translation”[SIC] [22]. In order to produce workshops that improve “productivity, innovation and translation” in the work of biomedical research professionals, the CIS has applied the principles of andragogy to team science education since its inception [22]. We use experiential learning methods to assess readiness to collaborate, promote participants’ self-reflection, balance didactics with interactive activities, ensure “hands-on” components, explicitly connect learning to practice, and draw on the wealth of team experience in each group of workshop attendees. Andragogy is a valuable learning theory that has been widely studied in other disciplines; however, it has not been applied to the field of team science education. Because we have found it to be one of the most valuable tools in our teaching approach, we examined the usefulness of integrating andragogical principles into team science training workshops, as evidenced by themes from the participants’ evaluation data. Given our emphasis on andragogical principles in educational practice, this work used an embedded study design with qualitative evaluation data as the primary focus, but with quantitative evaluation data also analysed to augment the qualitative data [23]. We undertook a phenomenological approach to investigate adult learners’ experiences of team science training through the lens of andragogy.

## Materials and Methods

### Participants and Study Setting

Team Science workshops from 2016 to 2021 were offered and promoted through a variety of email listservs, internal electronic newsletters, and websites aimed at UC research faculty and staff. Registration was voluntary for the majority of participants. A small minority were required to attend due to internal grant funding or training programme requirements. Workshops were primarily offered in an in-person setting and lasted 1–3 h. To accommodate COVID-19 restrictions, workshops were reformatted to be virtual and synchronous beginning in March 2020. The education team met weekly during this period to plan and deliver the workshops. Team meetings were multifaceted and included discussions on workshop logistics, content planning, slide and activity development, and a review of feedback from recent workshop evaluations. Importantly for this study, the education team also used these meetings to discuss andragogical principles, to ensure that several principles were incorporated into workshop planning, and to debrief on perceived successes and potential improvements of recent workshops, using both facilitator perspectives and learner evaluation feedback. This study was reviewed by our Institutional Review Board (IRB) and determined not to be human subject research (IRB #2024-0184).

### Data Collection

A paper evaluation survey was distributed to each participant at each in-person workshop, and participants were allotted time at the end of each workshop to complete it. Online workshop participants received an electronic evaluation survey link in the chat box at the conclusion of the workshop and via email shortly after the workshop ended. A minor update was made to the online survey instrument to include an additional question regarding the use of technology to achieve the goals of the virtual workshop. All evaluation surveys were anonymous and included Likert-scaled questions asking participants to rate the instructors, workshop content such as activities and examples, and the overall learning experience, as well as five open-ended questions. Although the evaluation instrument was not designed with andragogical principles in mind, these were incorporated into the workshop development process. The evaluation survey was initially developed for the purpose of improving educational programmes. However, the research team questioned whether the incorporation of andragogical theories into the educational programme was valuable for participants’ learning. The full evaluation survey instrument is provided in Supplementary Appendix SA.

This study used the qualitative data collected from responses to the open-ended questions of the instrument. We also selected a subset of the Likert-scaled questions that represented key andragogical principles. These included the value of the workshop in meeting one’s needs (Intrinsic Motivation), the Usefulness of handouts or other “takeaways” (Need to Know/Why), the Active involvement of participants in the learning experience (Self-Concept), the Use of practical examples (Readiness to Learn), and the Use of activities (Role of Experience, as workshop activities most often occur in small group discussions that focus on participant sharing and peer learning). Data from 6 years of workshop evaluations were combined into one dataset for analysis, with each evaluation marked as having occurred in-person or online.

### Data Analysis

A team of five qualitative coders used a deductive approach to analyse the evaluation survey data [24]. The research team applied andragogy as a theoretical framework both to analyse the data and to organise the study findings. Using a modified selective coding process, we sought data that supported the six principles of andragogy [25]. Although the evaluation instrument was not specifically designed with andragogy as a guiding framework, the analysis team sought to code learner responses to open-ended questions according to the principles of andragogy where appropriate and meaningful. Each coder analysed the data independently, and then the team met bimonthly to refine interpretations and resolve any differences between coders.

The research team recognised the difficulty of coding some participants’ comments as belonging to one theme or another. For instance, some comments were too short or too ambiguous to capture the underlying issue (e.g., “It was well done”), while other comments touched on multiple themes in the same sentence (e.g., “It was very interactive and engaging. Plus, it helps for people to think about how different people think and interact with each other”). Thus, the analysis team made decisions about how to interpret Knowles’ andragogy theory in the context of the workshop evaluation data and developed a codebook that included key words and phrases as examples of a particular theme to help facilitate our analysis [25]. The development of the codebook took place over several months of analysis team meetings in which each andragogical theme was reviewed and discussed, and both general and specific key words and phrases that correlated with a theme were identified. For example, comments that referred to a participant’s career stage or role in a team were coded under the theme “Readiness to Learn,” since this suggests that a learner’s readiness to learn is predicated on their social or professional role in life. Participants’ comments about activities that addressed specific problems (such as communication skills or team charters) and that utilised experiential learning that required learners to practise team science skills to solve a problem were coded under “Orientation to Learning,” an andragogical principle that relates to adults learning best through a problem-based approach. Fewer than 2% of participants’ comments were coded in multiple categories if the comment encompassed more than one theme (e.g., “Something less generic that I can actually apply to my team and current situation” has aspects of three themes: Readiness to Learn, Orientation to Learning, Need to Know/Why). More often a participant comment could be broken down into multiple themes by phrase or sentence (e.g., “Much more focused on ‘what can I do’ things and concepts. Take home messages much more tangible.” The first sentence of this comment was coded as Intrinsic Motivation, while the second sentence was coded as Orientation to Learning. Table 2 provides additional information on the key words and phrases that were incorporated into the codebook. This was essential to enabling the team to interpret the theoretical framework consistently within the specific context of the data set. Additionally, the data collected during the in-person workshops (2016-early 2020) were compared to the data collected during the virtual period (2020–21) to identify potential thematic differences that may have emerged as a result of this shift.

**TABLE 2:** Summary of the codebook for andragogical themes.

Principle	Keywords or phrases
1. Self-Concept	• Tools to take back to their teams
	• Tools to do on their own
	• More time for specific activities, discussions, or topics before moving on
	• Ability to direct activities, discussions, time allotment
2. Role of Experience	• More general need for discussion-based interactivity
	• Engaging with other participants
	• Sharing their own past experiences to learn from each other and build their knowledge
3. Readiness to Learn	• Different career stages/ages in a team
	• Training specific to their role in a team
4. Orientation to Learning	• Activities that address a specific problem (communication skills, charter)
	• Experiential learning, providing activities that require them to put TS principles into practice to address a problem
	• Hands-on activities
5. Need to Know/Why	• How is this information useful, valuable or beneficial to me?
	• Boring, too introductory, or impractical
	• Evidence-based
6. Intrinsic Motivation	• Self-improvement
	• Self-reflection

After deductively coding the evaluation data using andragogy as our theoretical framework, the analysis team inductively analysed the remaining data for themes that fell outside of the constructs of andragogy [24]. Quantitative data were summarised using means and standard deviations.

## Results

During the study period, 26 workshops were offered. Participation was voluntary for 23 workshops and required for 3 workshops as part of an institutional grant award. Workshop evaluations were collected anonymously; thus, individual participant demographics cannot be reported. In general, workshops included faculty, staff, and graduate students from UC, UC Medical Center, Cincinnati Children’s Hospital Medical Center (CCHMC), and a small number of members from outside the biomedical research community. In total, 363 evaluation surveys were received from participants in the 26 workshops.

Of the 363 evaluation surveys received, 605 unique pieces of feedback data were identified (i.e., individuals responded to more than one open-ended question and sometimes gave more than one response to individual questions). Approximately 85% of the comments provided by the participants were connected to Knowles’ theory of andragogy. All six of the themes of andragogy were present in the data set, although there were differences in frequency and emphasis on particular principles. The first five themes listed in Table 1 were the most frequently coded themes in the entire dataset, including self-directed learning, role of experience, readiness to learn, orientation to learning, and need to know/why. Intrinsic motivation was apparent in the evaluation data but to a much lesser extent.

Interestingly, participant responses to positive questions such as “why would you recommend this workshop to others?”, “what did you learn today that you are most likely to use in your work?”, and positive “other comments” were largely related to two themes: 1) participants’ readiness to learn based on their current professional roles or the roles of those they work with and 2) participants’ problem-based attitude to learning. Participant responses towards negative questions such as “why would you *not* recommend this workshop to others?”, “what suggestions for improvement do you have?”, “what were you hoping would be covered but was not?”, and negative “other comments” were most often connected to two different themes: 1) the role of experience and 2) the need for greater self-direction in their workshop experience. Participant responses coded under the need to know/why theme were more evenly spread throughout the evaluation data, with no consistent alignment with negative or positive questions. Deductive themes remained consistent across the complete data set for both in-person and online workshops.

The inductive analysis for themes unrelated to andragogy revealed one theme that participants greatly valued: meeting their biological needs for food, drink, and workshop breaks that allowed them to use the toilet or check in on their personal or professional business. This theme was particularly important in workshops that extended beyond 1.5 h. Given the shift to a virtual format in 2020, we reviewed the data for thematic changes between in-person and online workshops and this inductive theme was the only one no longer present. Table 3 provides a summary of each theme and representative quotes taken from the data set, with an approximate percentage of distribution within the dataset.

**TABLE 3:** Deductive and inductive themes with participant quotes and approximate percent representation and n in the data set (*N* = 605 responses).

Theme % Representation^a^ (n)	Participant Quotations
Self-Concept 13% (79)	“I expected more time for hands-on development of a charter; we had 15 min within roughly 1 h. Maybe send pre-work or use less time to lecture, which seemed very basic.”
	“Would have valued deeper engagement with topics, perhaps the follow-up topics.”
	“I’d recommend extending the time by about 30–45 min to allow for deeper engagement in group activities.”
Role of Experience 15% (91)	“Good ideas from others.”
	“Catering to expertise of audience. Have them lead discussions of best practices.”
	“More time for participants’ personal experiences.”
Readiness to Learn 16% (97)	“Workshop was helpful in learning about others’ work styles, which is helpful in harmonizing teams of collaborators.”
	“Possibly helpful suggestions for what to do depending on status within the team. I’m very young (grad student) and I think how I work/communicate in the team compared to senior members is very different.”
	“Allow teams to sit, work, talk, discuss together—my team leader and I came together and our time would have been better spent processing content together.”
Orientation to Learning 20% (121)	“More concrete suggestions and practical guidelines on how to manage team situations. Had good conversation about how these things are difficult. But what can we do to manage these issues?”
	“Share more examples of addressing dysfunctions in real life.”
	“More solutions and not just discussing problems.”
Need to Know/Why 10% (61)	“Practical and useful info.”
	“Go a bit more in-depth about scientific evidence which supports these concepts.”
	“Shorter, less fluffy, more data-driven. . .”
Intrinsic Motivation 4% (24)	“Much more focused on “what can I do” things and concepts.”
	“It helped to clarify my tendencies on a team.”
	“Recognizing my strengths/weakness and addressing them.”
Meeting Biological Needs^b^ 5% (30)	“Loved the coffee and lunches!”
	“Thanks for the coffee/breakfast and workshop!”
	“Thanks for the great info and snacks!”

^a^ Percentages do not sum to 100%; approximately 2% of data were coded to more than one deductive theme and the remaining 15% were not coded to either deductive or inductive themes.

^b^ Present in in-person workshop data only.

In the embedded mixed methods design, we analysed relevant quantitative data from the evaluation survey to determine whether it supported our qualitative findings. The quantitative data analysis showed that, when asked to rate aspects of the workshops that related to andragogical principles, participants mostly felt that the facilitators did a good-excellent job (scoring between 4 and 5 on a 5-point Likert scale) in addressing these needs. Table 4 provides the mean scores and standard deviations for the evaluation questions that are related to adult learning theory.

**TABLE 4:** Mean score and standard deviation for selected Likert scale questions (*N* = 363)^a^.

Survey item	Mean score (SD)
Value of the workshop in meeting your needs	4.2 (.36)
Usefulness of handouts or other “takeaways”	4.2 (.49)
Active involvement of participants in the learning experience	4.4 (.30)
Use of practical examples	4.3 (.35)
Use of activities	4.3 (.38)

^a^ 1–5 scale with 1 being Poor and 5 being Excellent.

The quantitative evaluation results support the qualitative findings by illustrating that participants felt that the team science workshops addressed key aspects of adult learning theory, and incorporated these aspects very well, based on high ratings in the good-excellent range.

## Discussion

The results indicate that andragogy is a useful and relevant learning theory to integrate into the development of effective team science training in a biomedical research setting. In this study, training effectiveness was measured by participant satisfaction, as evidenced by quantitative scoring of workshop components and qualitative feedback. These data also fill an important gap in the literature on learning theory as it relates to professional team functioning and the education of work teams in academic health. Adult learning theory has been explored in many disciplines; however, this study was the first to apply andragogy to team science training in an academic health centre through deductive analysis of workshop participant feedback.

Andragogy was well represented in the study data, with approximately 85% of participant evaluation feedback connecting to one or more of the six andragogical principles. Interestingly, participants commented positively on workshop aspects that addressed their readiness to learn and their problem-based attitude towards learning. When asked how to improve the workshop methods, the participants primarily requested teaching methods that addressed their desire for self-directed learning and the discussion of their own and others’ experiences as a learning resource. We speculate that this is because the workshop development team adequately addressed participants’ readiness to learn and problem-based learning orientation, but less so their need for self-direction and use of experience as expertise. Addressing adult learners’ desire for “why” they “need to know” information was a fifth theme that was widely represented in the data set, no matter the question type. Incorporating andragogical principles into training development and implementation is straightforward, requiring an awareness of adult learning principles and a process to ensure that “active learning” occurs during each educational event [26]. Study results demonstrate that learners respond positively to training designed with this theoretical framework in mind.

Our data suggest that the principles of andragogy are important to learners in our workshops; participants expressed their strengths and opportunities for improvement using language that fits within the themes of andragogy. Our findings suggest another important insight related to the application of andragogical principles to the field of team science training, which is that adults who pursue education to improve their work team functioning also value collaborative learning. Collaborative learning is an instructional approach that emphasises the pursuit of shared knowledge by both the instructor and the learners, while also asserting that learning and understanding are social in nature [27]. One study showed that social factors such as interacting with peers and instructors as part of the learning process improved learners’ academic performance [28]. Placing andragogy within a larger framework of collaborative learning expands the potential for learning in a complementary way, such that the team science workshop facilitator becomes a co-learner with the workshop participants. The facilitator determines the basic scaffolding to support learning; however, andragogical principles emerge as workshop participants help to direct the specifics of a discussion or learning experience, with both the facilitator and the participants co-creating knowledge as a collaborative team. There are several benefits to collaborative learning, some of which are uniquely suited to team science training because they inherently support several of the andragogical principles that were highly valued by participants in this study [27]. For example, in a collaborative learning environment, learners would be more active participants in the learning process to improve team functioning, and they would engage in self-directed, problem-based activities that rely heavily on learning from their own and other participants’ past experiences. By allowing learners to guide the direction of a workshop, they are likely to steer it towards content and discussions that are most useful and interesting to them, and to provide new information that addresses a need in their professional or personal lives, two other key principles of andragogy. However, in an increasingly hybrid or online learning environment, the use of technology to support active, collaborative learning presents challenges to instructors trying to meet the needs of adult learners [29].

Although present in the data set, intrinsic motivation was not a strong theme compared to the other five principles of andragogy. This may reflect the nature of the content of the team science training workshops, which certainly included self-assessment and encouraged self-reflection, but in the context of a team. Our evaluation survey may also lack questions that prompt comments on this motivation. Exploring intrinsic motivation in a different training setting that is more individual-focused, such as wellness seminars, courses aimed at improving technical skills, or leadership and management training might yield different results that suggest that internal motivation is equally important. Although it was not a major theme in this study, intrinsic motivation is an important andragogical principle that also aligns with the collaborative learning approach. Co-creation of educational activities and developing a shared understanding of team science topics would increase the likelihood of satisfying adult learners’ intrinsic motivation to learn.

### Strengths and Limitations of the Study

This study has many strengths and some limitations. First, it offers an analysis of the importance of andragogical principles in an educational field that was previously unexplored: team science. Using participant feedback as the primary source of data, this study demonstrates that adult learners express their training needs in ways that can be readily connected to andragogy, making it a useful learning theory for educators to consider when designing team science workshops. A limitation of this study is its focus on a training topic that is narrow in scope: team science training at an academic institution. Thus, generalisability to other institutions may be limited. Future research could expand the database to include evaluation data from team science training events at other institutions. Another possible limitation is the data set itself, which is bound to the written evaluation feedback provided by workshop participants. The data collection instrument was not originally designed with andragogy in mind, requiring the research team to identify and connect survey items to andragogical principles *post hoc*. Pre-hoc incorporation of questions specific to andragogy would likely allow for a more robust and comprehensive analysis of how important these principles are to team science trainees and what aspects of the workshop training were most successful in meeting the needs of adult learners. For example, the survey instrument could ask participants to rate how important each principle is to their individual learning, and the extent to which the workshop met each principle. Such targeted feedback would allow our workshop team to adjust content and instructional methods accordingly, increasing the likelihood of training success. Our data set did not include comments from all participants, nor did it include any other data source, such as longitudinal follow-up via survey or interview/focus group. We integrated quantitative data into an embedded study design in an effort to augment the qualitative findings. Finally, our primary method of analysis was deductive in nature, actively looking for themes that were corollary to the six principles of andragogy. Although we did employ an inductive analysis of any data that remained after the primary analysis, qualitative analyses that are deductive are limited by their nature.

The study results point to the importance of having a strong evaluation component in team science training programmes for continuous improvement that accommodates learner needs. Andragogy was found to be a valuable and relevant theoretical lens for interpreting adult learner feedback in a team science education and training context. Future studies that explore the relevance of collaborative learning principles to the needs of adult learners would be useful. Additionally, collecting participant feedback using an evaluation instrument that incorporates andragogy *a priori* may provide more explicit and robust data in relation to our research question.

## Conclusion

Since its development in the 1970s, andragogy has been applied to many fields of education and professional development. It is a useful and practical theoretical framework that can be applied to almost any adult learning experience, including team science training. Using direct participant feedback, our results show that andragogical principles are important in a biomedical research setting and that instructors should incorporate andragogy into the development and implementation of team science training opportunities in order to better meet the needs of adult learners. This work represents an advance in biomedical science because it demonstrates that andragogy can serve as a useful theoretical framework when designing team science training for biomedical researchers.

## Summary Table

### What Is Known About This Subject

• Malcolm Knowles developed andragogy theory to provide a framework for understanding the distinct learning patterns of adult learners.

• Andragogy has been demonstrated to be useful in several fields, but not in team science training for biomedical researchers.

### What This Paper Adds

• Using learner feedback, we evaluated the usefulness of integrating andragogy into team science training workshops.
• We found both quantitative and qualitative data suggesting that the incorporation of andragogical principles was valued by learners.
• This work represents an advance in biomedical science because it demonstrates that andragogy can serve as a useful theoretical framework when designing team science training for biomedical researchers.

## Data Availability

The raw data supporting the conclusion of this article will be made available by the authors, without undue reservation.
