# Supplementary material for: Andragogy in Practice: Applying a Theoretical Framework to Team Science Training in Biomedical Research
Source: Br J Biomed Sci. 2024 Mar 28;81:12651. doi: 10.3389/bjbs.2024.12651 (PMC11008574; doi:10.3389/bjbs.2024.12651)
Supplement: Supplementary file 1 [file DataSheet1.DOCX]

**Appendix A**

**Standard Workshop Evaluation Survey**

**Workshop Evaluation**

**Date:**

**Workshop Title:**

How would you rate the following?

| **Poor** | **Fair** | **Average** | **Good** | | **Excellent** | | | | |
| --- | --- | --- | --- | --- | --- | --- | --- | --- | --- |
| **1** | **2** | **3** | **4** | | **5** | | | | |
|  | | | | **1** | | **2** | **3** | **4** | **5** |
| 1. Value of workshop in meeting your needs | | | |  | |  |  |  |  |
| 1. Expertise of the presenter(s) | | | |  | |  |  |  |  |
| 1. Presentation techniques of the presenter(s) | | | |  | |  |  |  |  |
| 1. Your learning experience overall | | | |  | |  |  |  |  |
| 1. Usefulness of handouts or other “take-aways” | | | |  | |  |  |  |  |
| 1. Clarity of objectives | | | |  | |  |  |  |  |
| 1. Active involvement of participants in learning experience | | | |  | |  |  |  |  |
| 1. Timeliness of the material presented | | | |  | |  |  |  |  |
| 1. Use of practical examples | | | |  | |  |  |  |  |
| 1. Use of activities | | | |  | |  |  |  |  |
| 1. Use of technology in a virtual environment | | | |  | |  |  |  |  |
| 1. Overall rating of the session | | | |  | |  |  |  |  |

Would you recommend this workshop to others? Yes __ No __

Why?

What suggestions for improvement do you have?

What did you learn today that you are will most likely use in your work?

What were you hoping would be covered, but was not?

Other comments?
